# Postoperative Outcomes After Rectal Cancer Surgery With or Without Primary Anastomosis: A Propensity Score–Weighted Study

**DOI:** 10.3390/diagnostics16101533

**Published:** 2026-05-19

**Authors:** Nicoleta Aurelia Sanda, Petruta Violeta Filip, Florin Teodor Bobirca, Andreea-Nicoleta Marinescu, Alexandru Chirca, Daniela Aurora Peșu, Roxana Florina Ristea, Radu Virgil Costea

**Affiliations:** 1 Faculty of Medicine, University of Medicine and Pharmacy “Carol-Davila”, 020021 Bucharest, Romania; nicoleta.sanda@umfcd.ro (N.A.S.); petruta.filip@umfcd.ro (P.V.F.); alexandru.chirca@umfcd.ro (A.C.); radu.costea@umfcd.ro (R.V.C.); 2 University Emergency Hospital Bucharest, 050098 Bucharest, Romania; daniela.pesu@umfcd.ro; 3 Surgical Department I, “Dr. I. Cantacuzino” Clinical Hospital, 030167 Bucharest, Romania; 4 Surgical Department, Medicover Hospital, 020332 Bucharest, Romania; roxristea@yahoo.com

**Keywords:** rectal cancer, primary anastomosis, propensity score, overlap weighting, postoperative complications, anastomotic leak, observational study

## Abstract

**Background:** The role of primary anastomosis in rectal cancer surgery remains debated, particularly due to concerns regarding postoperative morbidity. Evidence from randomized trials is limited, and observational studies are frequently affected by selection bias. **Methods:** We conducted a retrospective observational study including patients undergoing rectal cancer surgery with or without primary anastomosis. To reduce confounding, propensity scores were estimated using relevant clinical and oncologic covariates, and overlap weighting was applied to estimate treatment effects in a population with clinical equipoise. The primary outcome was any postoperative complication. Secondary outcomes included severe postoperative complications, reintervention, in-hospital mortality, and measures of healthcare resource utilization. Absolute risk differences (RDs) and mean differences (MDs) with 95% confidence intervals (CIs) were calculated. Stabilized inverse probability of treatment weighting was used as a sensitivity analysis. **Results:** A total of 173 patients were included. After overlap weighting, primary anastomosis was not associated with a statistically significant difference in overall postoperative complications compared with no anastomosis (RD +0.01, 95% CI −0.14 to +0.17). Severe postoperative complications were numerically more frequent in the primary anastomosis group, while reintervention rates were numerically lower; however, these differences did not reach statistical significance. In-hospital mortality was significantly lower among patients undergoing primary anastomosis (RD −0.08, 95% CI −0.16 to −0.02). No significant differences were observed in length of hospital stay or intensive care unit utilization. **Conclusions:** Postoperative outcomes after rectal cancer surgery appeared broadly comparable between patients managed with or without primary anastomosis after adjustment for measured baseline characteristics. These findings should be interpreted in the context of residual confounding and surgical selection, and support individualized decision-making rather than routine use of either restorative or non-restorative strategy.

## 1. Introduction

Rectal cancer surgery frequently requires intraoperative decisions regarding restoration of bowel continuity, particularly in patients with advanced disease or low rectal tumors [1,2]. While primary anastomosis offers the potential benefits of preserved intestinal continuity and avoidance of a permanent stoma, it is also associated with an increased risk of postoperative complications, most notably anastomotic leak [3,4]. As a result, the decision to perform primary anastomosis is highly individualized and influenced by multiple patient-, tumor-, and procedure-related factors [5]. In very low rectal cancer, sphincter-preserving strategies such as intersphincteric resection further highlight the complexity of the anastomotic decision-making process and the balance between oncologic safety and functional outcomes [6,7].

Anastomotic leak remains one of the most feared complications following rectal cancer surgery and represents a major determinant of surgical decision-making [4,8]. Even when not all patients undergo intestinal reconstruction, the anticipated risk of leak often guides the choice between primary anastomosis and alternative strategies, including procedures such as Hartmann’s operation [9,10]. Importantly, because anastomosis is not performed uniformly across patients, leak cannot be meaningfully compared as a postoperative outcome between all surgical groups, underscoring the need for careful interpretation of comparative analyses. Therefore, comparative analyses should focus on outcomes applicable to both strategies (overall morbidity, severe complications, reintervention, and in-hospital mortality). Existing observational studies comparing rectal cancer surgery with and without primary anastomosis have reported heterogeneous results regarding postoperative morbidity and mortality [11,12,13,14]. These inconsistencies are largely attributable to substantial baseline differences between patient groups, as individuals selected for primary anastomosis are typically younger, have fewer comorbidities, and present with more favorable tumor characteristics [13,14,15]. Conventional unadjusted comparisons therefore risk confounding by indication and may overestimate or underestimate the true association between surgical strategy and postoperative outcomes. In clinical practice, the decision to perform a diverting stoma represents an additional strategy to mitigate the clinical consequences of anastomotic leak, particularly in low rectal anastomoses [14].

Propensity score-based methods offer a robust approach to address these limitations in observational research [16]. In particular, overlap weighting emphasizes patients with comparable probabilities of receiving either treatment strategy, thereby approximating the conditions of a randomized comparison while retaining the clinical realism of observational data [17,18,19]. This approach is especially relevant in rectal cancer surgery, where treatment assignment is strongly driven by clinical judgment rather than random allocation.

The aim of this study was to evaluate postoperative outcomes following rectal cancer surgery with or without primary anastomosis using propensity score-based overlap weighting to account for baseline clinical differences [19]. In addition to comparative analyses of postoperative morbidity and mortality, anastomotic leak was assessed descriptively among patients undergoing primary anastomosis to provide clinically relevant context regarding its incidence, severity, and postoperative burden. Overlap weighting was selected because it focuses the analysis on patients with clinical equipoise and reduces the influence of extreme propensity scores, thereby improving covariate balance and the stability of treatment effect estimates.

## 2. Methods

### 2.1. Study Design and Patient Selection

This retrospective observational study included consecutive patients undergoing curative-intent surgery for rectal cancer at a tertiary referral center. Patients were identified from a prospectively maintained institutional database. Rectal cancer was defined as a tumor located within 15 cm from the anal verge, as assessed by preoperative imaging or intraoperative findings [1,20].

Patients were eligible for inclusion if they underwent elective or urgent rectal resection with or without primary anastomosis. Exclusion criteria included palliative procedures, local excisions, and incomplete surgical records precluding outcome assessment.

The final analytical cohort comprised 173 patients with complete data for all primary postoperative outcomes. Patients were classified according to whether a primary anastomosis was performed at the time of surgery. Classification was based on the recorded presence or absence of a primary anastomosis in the operative dataset. The anastomosis group included restorative procedures, such as low and ultralow anterior resections with colorectal or coloanal anastomosis. Although detailed classification according to specific surgical procedures (e.g., low vs ultralow anterior resection) was not uniformly available, anastomotic level was recorded and used as a surrogate for surgical stratification. This allowed clinically meaningful grouping into low (≤5 cm), mid (6–10 cm), and high (>10 cm) anastomoses, which reflect differences in surgical complexity and risk. Patients without anastomosis included non-restorative procedures, such as Hartmann-type operations and stoma-forming procedures.

The decision to perform a primary anastomosis was based on intraoperative clinical judgment, taking into account multiple patient-related, tumor-related, and technical factors. As a result, treatment allocation was inherently non-random and subject to confounding by indication. To address this limitation, propensity score-based methods were applied to reduce imbalance in observed baseline characteristics between groups. All surgical procedures were performed using an open approach. Information regarding the use of protective stomas (e.g., diverting ileostomy) was not systematically available in the dataset.

### 2.2. Exposure and Outcomes

The exposure of interest was performance of a primary anastomosis at the time of rectal cancer surgery. Patients were categorized into two groups according to whether a primary anastomosis was performed or not.

The primary outcome was any postoperative complication. Secondary outcomes included severe postoperative complications (Clavien–Dindo grade ≥ III), in-hospital mortality, and reintervention [21]. Continuous outcomes included length of hospital stay and intensive care unit stay.

Anastomotic leak was evaluated descriptively among patients undergoing primary anastomosis only. Anastomotic leak was diagnosed based on clinical findings and imaging studies (primarily computed tomography), in accordance with routine clinical practice [4]. Overall leak incidence was recorded, and a clinically significant leak was defined as leak requiring surgical reintervention, corresponding to severe (grade C) events. Duration of leak was recorded in days and analyzed in the subset of patients with anastomotic leak. Radiologic or endoscopic interventions were not systematically recorded in the dataset and were therefore not included in the definition of clinically relevant leak.

### 2.3. Covariates

Baseline covariates were selected a priori based on clinical relevance and potential association with both treatment assignment and postoperative outcomes, reflecting patient-related, tumor-related, and procedure-related factors. These included age, sex, ASA class, Charlson Comorbidity Index, pathological tumor stage, tumor height from the anal verge, receipt of neoadjuvant therapy, urgency of surgery (elective vs. emergency), and type of surgical procedure [22,23].

### 2.4. Propensity Score Model and Weighting

To address baseline differences between patients undergoing rectal cancer surgery with or without primary anastomosis, propensity score-based methods were applied. The propensity score represented the probability of receiving a primary anastomosis conditional on observed baseline covariates.

The propensity score model included clinically relevant variables selected a priori based on surgical judgment and prior literature. Covariates comprised age, sex, ASA physical status (ASA), Charlson Comorbidity Index, pathological tumor stage, tumor height from the anal verge, receipt of neoadjuvant therapy, urgency of surgery, and type of surgical procedure [22,23]. Variables were chosen to capture patient-related, tumor-related, and procedural factors influencing the decision to perform a primary anastomosis [16,17].

ASA physical status was modeled as an ordinal variable, and pathological tumor stage as a categorical variable.

Overlap weighting was used as the primary analytical approach. This method assigns greater weight to patients with a higher probability of receiving either treatment strategy, thereby emphasizing the region of common clinical equipoise and improving covariate balance between groups. Covariate balance before and after weighting was assessed using standardized mean differences, with values below 0.1 indicating adequate balance [24].

As a sensitivity analysis, stabilized inverse probability of treatment weighting targeting the average treatment effect (ATE) was also performed to evaluate the robustness of the findings across alternative weighting strategies [25].

### 2.5. Statistical Analysis

Baseline characteristics were summarized using medians with interquartile ranges for continuous variables and counts with percentages for categorical variables. Comparisons of baseline characteristics were descriptive only and not subjected to hypothesis testing.

Treatment effects for binary postoperative outcomes were estimated as risk differences (RDs) using propensity score-based overlap weighting as the primary analytical approach. This weighting strategy was used to improve balance in measured baseline covariates while focusing the analysis on patients with comparable probabilities of receiving either treatment. Confidence intervals were calculated using robust variance estimation to account for weighting. Results are reported as RD with 95% confidence intervals [16,17,18].

Continuous outcomes were analyzed using weighted mean differences. For these analyses, available-case analysis was performed, and the effective sample size varied according to data completeness for each outcome.

As a sensitivity analysis, stabilized inverse probability of treatment weighting targeting the average treatment effect (ATE) was applied, and results were compared qualitatively with the primary overlap-weighted estimates to assess robustness [25].

Missing data were handled by excluding patients with incomplete information from the respective analyses only; no imputation was performed. All analyses were conducted using R (R Foundation for Statistical Computing, Vienna, Austria). All statistical analyses were conducted by members of the research team with formal training in biostatistics and experience in clinical research.

## 3. Results

### 3.1. Study Population

A total of 173 patients undergoing rectal cancer surgery were included in the final analysis. Of these, 72 underwent primary anastomosis and 101 underwent surgery without intestinal reconstruction, according to intraoperative clinical judgment. Baseline demographic, clinical, and oncologic characteristics of the study population are summarized in Table 1.

Before weighting, patients undergoing primary anastomosis differed from those without anastomosis with respect to several baseline characteristics, reflecting the non-random nature of treatment allocation. In particular, differences were observed in measures of baseline physiological status, comorbidity burden, tumor stage, and urgency of surgery.

### 3.2. Covariate Balance After Propensity Score Weighting

**Figure 1:**
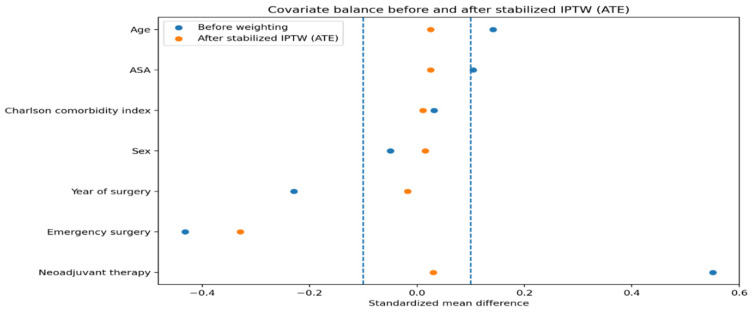
Covariate balance before and after stabilized inverse probability of treatment weighting (IPTW, ATE). Absolute standardized mean differences (SMDs) for baseline covariates are shown before weighting and after application of stabilized inverse probability of treatment weighting targeting the average treatment effect (ATE). Dashed vertical lines indicate the prespecified threshold of |SMD| = 0.1, below which covariate balance is considered adequate.

Application of propensity score-based weighting resulted in substantial improvement in covariate balance between the two treatment groups. After stabilized inverse probability of treatment weighting, standardized mean differences for all predefined baseline covariates were below the prespecified threshold of 0.1 (Figure 1). Overlap weighting achieved similarly excellent covariate balance and was used for the primary outcome analyses.

**Figure 2:**
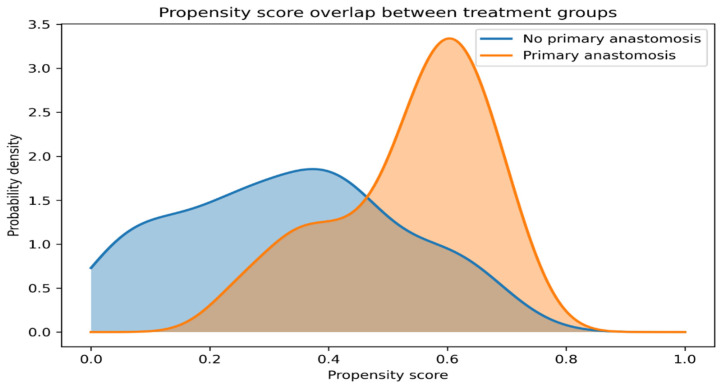
Propensity score overlap between treatment groups. Kernel density distributions of propensity scores for patients undergoing primary anastomosis and those without anastomosis demonstrate adequate overlap, supporting the use of overlap weighting.

These results support the suitability of the propensity score–weighted cohort for subsequent comparative analyses of postoperative outcomes. Distributions of propensity scores for patients undergoing primary anastomosis and those without anastomosis demonstrated adequate overlap between treatment groups, supporting the use of propensity score-based weighting approaches, including overlap weighting, for comparative analyses (Figure 2). All covariates included in the propensity score model are reported as baseline characteristics in Table 1.

### 3.3. Postoperative Outcomes

#### 3.3.1. Primary and Secondary Binary Outcomes

Postoperative outcomes after propensity score weighting are summarized in Table 2. After application of overlap weighting, no statistically significant difference was observed in the overall risk of any postoperative complication between patients undergoing primary anastomosis and those without anastomosis (RD +0.01, 95% CI −0.14 to +0.17).

Severe postoperative complications (Clavien–Dindo grade ≥ III) occurred numerically more frequently in the primary anastomosis group, although the absolute risk difference did not reach statistical significance (RD +0.05, 95% CI 0.00 to +0.12). Rates of reintervention were numerically lower among patients undergoing primary anastomosis, but this difference was not statistically significant (RD −0.07, 95% CI −0.15 to +0.02).

In-hospital mortality was lower in the primary anastomosis group, with a statistically significant absolute risk reduction observed after overlap weighting (RD −0.08, 95% CI −0.16 to −0.02). Sensitivity analyses using stabilized inverse probability of treatment weighting yielded consistent results across all binary outcomes (Table 2).

#### 3.3.2. Healthcare Resource Utilization

Analyses of healthcare resource utilization are presented in Table 3. After overlap weighting, patients undergoing primary anastomosis had a numerically shorter length of hospital stay compared with those without anastomosis; however, this difference did not reach statistical significance (MD −4.0 days, 95% CI −9.45 to +1.37).

No meaningful difference was observed in intensive care unit length of stay between the two groups (MD +0.01 days, 95% CI −0.34 to +0.36). Sensitivity analyses using stabilized inverse probability weighting showed similar results for both outcomes (Table 3).

#### 3.3.3. Anastomotic Leak

Anastomotic leak was evaluated descriptively among patients undergoing primary anastomosis. Because intestinal reconstruction was not performed in all patients, anastomotic leak was not analyzed as a comparative outcome between treatment groups.

Anastomotic leak showed a clinically meaningful gradient according to anastomotic level, with both incidence and postoperative burden decreasing as the anastomosis was located further from the anal verge. Lower anastomoses were associated with higher leak rates and longer leak duration, whereas leaks were less frequent and shorter in duration for higher anastomoses. Clinically relevant leaks were observed only in patients with mid-level anastomoses.

Detailed descriptive data on anastomotic leak incidence, severity, and duration according to anastomotic level are provided in Table 4.

#### 3.3.4. Additional Analyses and Robustness

Distributions of propensity scores and analysis weights demonstrated adequate overlap between treatment groups and stable weight behavior, supporting the robustness of the propensity score–weighted analyses.

**Figure 3:**
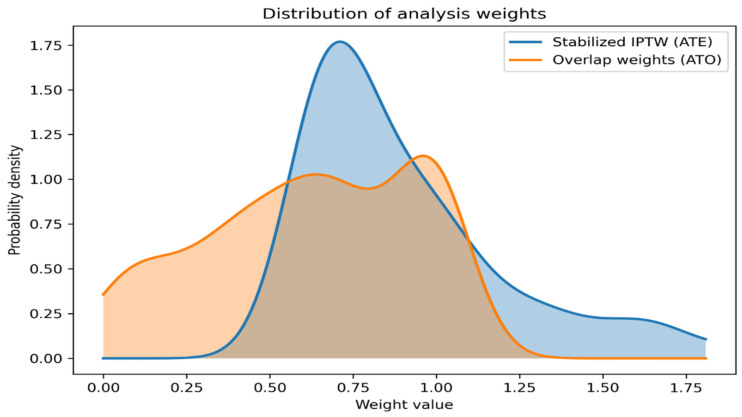
Distribution of analysis weights. Kernel density distributions of stabilized inverse probability of treatment weights (ATE) and overlap weights (ATO) are shown. Overlap weighting yields a more concentrated weight distribution, whereas stabilized IPTW shows greater dispersion.

Results of sensitivity analyses using stabilized inverse probability of treatment weighting (ATE) were consistent with the primary overlap-weighted analyses across all evaluated outcomes. Distributions of analysis weights showed a more concentrated pattern for overlap weights compared with stabilized inverse probability weights, indicating stable weight behavior and limited influence of extreme observations (Figure 3).

## 4. Discussion

In this propensity score–weighted observational study, we evaluated postoperative outcomes after rectal cancer surgery performed with or without primary anastomosis. After adjustment for baseline differences using overlap weighting, primary anastomosis was not associated with a reduction in overall postoperative complications. While severe complications occurred numerically more often in the anastomosis group and reintervention rates were numerically lower, these differences did not reach statistical significance. In-hospital mortality was lower among patients undergoing primary anastomosis.

The absence of a significant difference in overall postoperative morbidity highlights the complexity of surgical decision-making in rectal cancer [26]. In routine surgical practice, non-restorative procedures such as Hartmann-type operations are often preferentially selected for patients perceived to have higher operative risk [10]. These include patients with greater comorbidity burden, technically challenging pelvic anatomy, lower tumors, prior neoadjuvant treatment, or unfavorable intraoperative conditions. Although overlap weighting substantially improved balance across measured covariates, important determinants of surgical judgment remain difficult to fully capture in retrospective datasets. Importantly, the apparent balance achieved in the weighted groups should not be interpreted as indicating that the original treatment groups were clinically similar. Rather, it reflects the intended effect of propensity score weighting, which reduces imbalance in measured baseline characteristics and allows comparison among patients with overlapping clinical profiles. Therefore, the comparable postoperative morbidity observed in the weighted cohort should be interpreted as reflecting outcomes among clinically selected patients rather than as evidence of equivalence between restorative and non-restorative strategies. This finding should not be interpreted as indicating unreliable surgical decision-making; instead, it may reflect risk-adapted selection of the operative strategy in patients with different baseline and intraoperative risk profiles.

The observed reduction in in-hospital mortality among patients undergoing primary anastomosis should be interpreted with caution. In routine clinical practice, patients selected for primary anastomosis often have a more favorable baseline profile, including better physiological reserve, fewer comorbidities, and less locally advanced disease [11,13]. However, the decision to perform an anastomosis is also influenced by intraoperative and technical factors that are not fully captured by structured baseline variables. These include tissue quality, tissue perfusion, anastomotic tension, precise pelvic anatomy, and the surgeon’s assessment of reconstructive safety. Although overlap weighting achieved excellent balance across measured covariates, these unmeasured factors may still contribute to residual confounding. Therefore, the lower in-hospital mortality observed in the primary anastomosis group is more plausibly attributable to residual surgical selection bias than to a direct protective effect of primary anastomosis.

Healthcare resource utilization outcomes further support a nuanced interpretation of the results. Length of hospital stay was numerically shorter among patients undergoing primary anastomosis, although this difference was not statistically significant, and no meaningful difference was observed in ICU utilization. These findings suggest that primary anastomosis, when appropriately selected, does not substantially increase postoperative resource use, but also does not guarantee shorter recovery in all patients.

Anastomotic leak remains one of the most feared complications following rectal cancer surgery and plays a central role in intraoperative decision-making [4,8,27]. Beyond its immediate postoperative impact, leak may also affect longer-term oncologic outcomes [28]. Although leak cannot be meaningfully compared between patients with and without primary anastomosis, its anticipated risk strongly influences the choice between restoration of bowel continuity and avoidance of an anastomosis. For this reason, descriptive evaluation of leak characteristics provides important clinical context for interpreting postoperative outcomes.

In the present study, anastomotic leak demonstrated a clinically meaningful gradient according to anastomotic level, with higher leak incidence and longer leak duration observed in lower anastomoses. This pattern is consistent with previous reports identifying anastomotic height as an important determinant of leak risk and severity in rectal cancer surgery [29,30,31,32]. From a technical perspective, low rectal reconstruction is particularly challenging in patients with a narrow male pelvis, fibrosis following neoadjuvant chemoradiotherapy, and ultralow anastomoses requiring difficult pelvic dissection and reconstruction [6,7]. These factors may increase anastomotic tension, compromise tissue perfusion, and limit access for safe stapling or suturing, thereby contributing to leak risk and postoperative morbidity.

Notably, although leak incidence was higher in lower anastomoses, no grade C events were observed in the lowest anastomotic group (≤5 cm), suggesting that not all leaks translated into clinically severe complications. A clinically significant leak was defined as leak requiring surgical reintervention, corresponding to severe (grade C) events. According to the International Study Group of Rectal Cancer (ISREC) classification, clinically relevant leaks also include grade B events managed without reoperation [4]. Because conservative management strategies, such as radiologic or endoscopic interventions, were not systematically captured in our dataset, grade B leaks may be underrepresented [33]. As a result, our analysis primarily reflects the burden of more severe leak events. This approach focuses on clinically consequential complications with greater postoperative impact. Reporting the duration of leak offers additional insight into the postoperative burden associated with this complication, a dimension that is infrequently addressed in observational studies. Together, these descriptive findings underscore the rationale for a selective and individualized approach to primary anastomosis, particularly in patients with low rectal tumors.

Our study has several strengths. The use of overlap weighting allowed us to focus on a population with clinical equipoise and to achieve excellent covariate balance, as demonstrated by standardized mean differences below accepted thresholds [24,34]. This approach reduces the influence of patients with extreme treatment probabilities and enhances the interpretability of treatment effect estimates. In addition, we reported absolute risk and mean differences, which provide clinically meaningful measures of effect size and facilitate decision-making at the bedside.

Several limitations should be acknowledged. First, the observational design precludes causal inference, and residual confounding remains possible despite rigorous adjustment. Second, this was a single-center study conducted in a tertiary referral center, which may limit the generalizability of the findings. Other practice settings may differ in patient populations, healthcare systems, organizational characteristics, healthcare resource utilization, and regional cancer outcome patterns [12,35]. Third, missing data were present for selected secondary outcomes related to healthcare utilization; however, all primary postoperative outcomes were complete, and analyses of continuous outcomes were transparently conducted on an available-case basis.

In addition, systematic data regarding diverting ileostomy usage were not available. This represents an important limitation, because fecal diversion may substantially influence both the clinical severity of anastomotic leak and subsequent postoperative outcomes [14]. Consequently, the present analysis could not account for the potential modifying effect of diversion on leak-related morbidity.

The use of in-hospital mortality instead of standardized 30-day or 90-day mortality may limit comparability with other studies. Furthermore, although composite measures such as the Comprehensive Complication Index (CCI) may provide a more nuanced assessment of postoperative morbidity, such metrics were not available in our dataset. However, the use of clinically meaningful binary outcomes remains widely accepted in surgical outcome research. Finally, longer-term oncologic outcomes were not the focus of this analysis and were therefore not evaluated comparatively [28].

Taken together, our findings support a selective rather than routine use of primary anastomosis in rectal cancer surgery. The decision to perform an anastomosis should remain individualized, integrating patient fitness, disease characteristics, and intraoperative assessment, rather than being driven by an expectation of uniformly improved short-term outcomes. Longer-term oncologic outcomes were beyond the scope of this analysis and warrant further investigation in dedicated studies.

## 5. Conclusions

In this propensity score–weighted observational study, postoperative outcomes after rectal cancer surgery appeared broadly comparable between patients undergoing surgery with or without primary anastomosis after adjustment for measured baseline characteristics. These findings should be interpreted cautiously, as residual confounding and surgical selection bias related to intraoperative decision-making likely persist despite weighting. Rather than supporting routine use of either restorative or non-restorative strategy, the present results reinforce the importance of individualized surgical decision-making based on patient fitness, tumor characteristics, technical feasibility, and intraoperative assessment.

Anastomotic leak, assessed descriptively among patients undergoing primary anastomosis, showed a clinically meaningful relationship between anastomotic level and postoperative burden, particularly in low rectal reconstruction. This finding further highlights the importance of careful anastomotic planning and individualized risk assessment, especially in patients with technically challenging pelvic anatomy.

Future studies incorporating multicenter data, systematic information on diverting stoma use, standardized leak severity, and longer-term outcomes may further clarify the role of primary anastomosis across different clinical contexts.

## Figures and Tables

**Table 1:** Baseline clinical and oncologic characteristics of patients undergoing rectal cancer surgery with and without primary anastomosis (*N* = 173).

Characteristic	Overall	No Anastomosis	Primary Anastomosis
ASA score	3 (2–3)	3 (2–3)	3 (3–3)
Age, years	63 (57–72)	62 (57–71)	64 (57–72)
Charlson comorbidity index	5 (4–7)	5 (4–7)	6 (4–7)
Emergency surgery	9/169 (5.3%)	9/98 (9.2%)	0/71 (0.0%)
Neoadjuvant therapy	51/173 (29.5%)	19/101 (18.8%)	32/72 (44.4%)
Sex: Female	72/173 (41.6%)	41/101 (40.6%)	31/72 (43.1%)
Sex: Male	101/173 (58.4%)	60/101 (59.4%)	41/72 (56.9%)
Tumor stage: 0	3/155 (1.9%)	3/83 (3.6%)	0/72 (0.0%)
Tumor stage: 1	31/155 (20.0%)	15/83 (18.1%)	16/72 (22.2%)
Tumor stage: 2	54/155 (34.8%)	21/83 (25.3%)	33/72 (45.8%)
Tumor stage: 3	45/155 (29.0%)	27/83 (32.5%)	18/72 (25.0%)
Tumor stage: 4	22/155 (14.2%)	17/83 (20.5%)	5/72 (6.9%)
Tumor height from anal verge (cm)	7 (5–10)	6 (5–9.5)	8 (5–10)

Notes: Values are presented as median (interquartile range) for continuous variables and *n* (%) for categorical variables. Percentages are calculated using available data for each variable. Abbreviations: ASA, American Society of Anesthesiologists.

**Table 2:** Absolute risk differences (RDs) with 95% confidence intervals (CIs) for postoperative outcomes comparing primary anastomosis with no anastomosis, estimated using overlap weighting (ATO) and stabilized inverse probability of treatment weighting (ATE).

Outcome	RD (ATO)	95% CI	RD (ATE)	95% CI	*N*
Any postoperative complication	+0.012	−0.137 to +0.167	+0.023	−0.120 to +0.170	173
Severe complications (Clavien–Dindo ≥ III)	+0.054	+0.000 to +0.119	+0.057	+0.000 to +0.128	173
Reintervention	−0.070	−0.154 to +0.015	−0.057	−0.137 to +0.033	173
Mortality	−0.083	−0.158 to −0.018	−0.078	−0.143 to −0.020	173

Notes: Risk differences (RDs) represent absolute differences in outcome risk between patients undergoing primary anastomosis and those managed without an anastomosis. Positive RD values indicate higher risk associated with primary anastomosis.

**Table 3:** Mean differences (MDs) with 95% confidence intervals (CIs) for continuous postoperative outcomes comparing primary anastomosis with no anastomosis, estimated using overlap weighting (ATO) and stabilized inverse probability of treatment weighting (ATE).

Outcome	Mean (ATO) Primary Anastomosis (*n* = 72)	Mean (ATO) No Anastomosis (*n* = 101)	MD (ATO)	95% CI (ATO)	MD (ATE)	95% CI (ATE)	*N*
Length of hospital stay, days	25.91	29.96	−4.04	−9.45 to +1.37	−2.92	−7.48 to +1.64	138
ICU stay, days	0.19	0.18	+0.01	−0.34 to +0.36	−0.24	−0.82 to +0.34	136

Notes: Mean differences (MDs) represent absolute differences in mean values between patients undergoing primary anastomosis and those managed without an anastomosis. Negative MD values indicate shorter duration associated with primary anastomosis. Overlap weighting (ATO) was used for the primary analysis, with stabilized inverse probability of treatment weighting (ATE) performed as a sensitivity analysis. *N* reflects the number of patients with available data for each outcome. Abbreviations: ATE, average treatment effect; ATO, overlap weighting; CI, confidence interval; ICU, intensive care unit; MD, mean difference.

**Table 4:** Anastomotic leak incidence, severity, and duration according to anastomotic level among patients undergoing primary anastomosis.

Anastomotic Level (cm)	Patients (*n*)	Leak Incidence	Clinically Relevant Leak	Leak Burden
		Any leak, *n* (%)	Leak with reintervention, *n* (%)	Duration, days (median, IQR)
≤5 cm	22	7 (31.8%)	0 (0.0%)	11 (10–12)
6–10 cm	35	8 (22.9%)	1 (2.9%)	11 (10–15)
>10 cm	13	1 (7.7%)	0 (0.0%)	8 (8–8)

Notes: Anastomotic leak was evaluated descriptively among patients undergoing primary anastomosis only. A clinically relevant leak was defined by the need for surgical reintervention. Duration of leak reflects the postoperative burden among patients with documented leak.

## Data Availability

The data that support the findings of this study are available from the corresponding author upon reasonable request.
